# The transformative potential of artificial intelligence in pediatric medicine: Current applications, methodological challenges, and future directions

**DOI:** 10.1002/ped4.70061

**Published:** 2026-05-01

**Authors:** Ruisong Wang, Xiaoman Ding, Wanyue Zhang, Tieliu Shi

**Affiliations:** ^1^ College of Medicine Hunan University of Arts and Science Changde Hunan China; ^2^ The Center for Bioinformatics and Computational Biology, Shanghai Key Laboratory of Regulatory Biology, Institute of Biomedical Sciences and School of Life Sciences East China Normal University Shanghai China; ^3^ Key Laboratory of Advanced Theory and Application in Statistics and Data Science ‐ MOE East China Normal University Shanghai China; ^4^ College of Life Science and Technology Xinjiang University Urumqi Xinjiang China

**Keywords:** Artificial intelligence, Deep learning, Machine learning, Pediatrics, Precision medicine

## Abstract

Artificial intelligence (AI) possesses the transformative potential to reshape pediatric medicine, offering powerful tools for diagnosis, prognosis, and personalized therapy. This review focuses on three domains selected for their relative maturity in AI development and proximity to clinical translation—pediatric critical care, perinatal and neonatal medicine, and precision oncology—evaluating current evidence for clinical utility and outlining challenges to implementation. AI is demonstrating significant potential across these domains: in critical care, deep learning models outperform traditional scoring systems for dynamic prediction of adverse events; in perinatal and neonatal medicine, AI enhances prenatal ultrasonography and integrates multiomics data to guide complex therapies; and in oncology, radiomics, and genomic analysis enable non‐invasive tumor characterization and personalized treatment strategies. However, significant hurdles remain. Foundational data challenges—including scarcity, heterogeneity, and limited sharing of pediatric data—are being addressed through transfer learning, federated learning, and synthetic data generation. Clinical translation is further impeded by algorithmic bias, the ‘black box’ problem, and the unique developmental physiology of children, which demands age‐specific model validation. Future progress depends on multi‐institutional collaboration, a research focus that extends beyond prediction to encompass causal inference and explainability, and the establishment of robust ethical, regulatory, and economic frameworks. Ultimately, responsible implementation of AI in pediatrics requires building systems that are not merely accurate but transparent, equitable, and trustworthy.

## Graphical abstract

Artificial intelligence (AI) offers transformative potential for paediatric diagnosis and treatment, yet implementation faces unique challenges, including data scarcity, algorithmic bias, and children's developmental physiology. This review examines current applications and charts a path toward transparent, equitable, and trustworthy AI in child health.

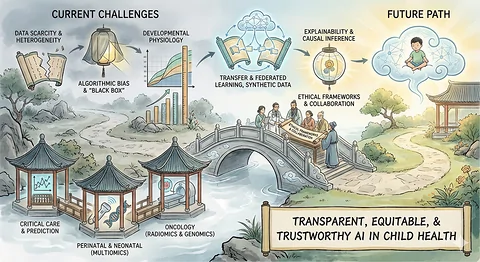

## INTRODUCTION

### The pediatric imperative

The practice of pediatric medicine is defined by its unique complexities. It encompasses a patient population undergoing continuous physiological, metabolic, and neurological development from the prenatal period to adolescence, indicating that disease presentation, progression, and therapeutic response vary dramatically with age. Consequently, clinical models derived from adult data may be inappropriate or even harmful when applied to children.^1^, ^2^, ^3^ This challenge is amplified in the data‐driven era; a recent systematic review demonstrated that the underrepresentation of children in public medical datasets risks entrenching age‐related bias in biomedical artificial intelligence (AI), potentially compelling clinicians towards the ‘off‐label’ application of adult‐trained algorithms.^4^

Beyond this developmental variability, the pediatric disease landscape is further complicated by a preponderance of rare conditions and multifactorial etiologies. Congenital heart disease (CHD) exemplifies this complexity: despite advances in genetic testing, a definitive etiology remains unidentified in approximately 60%–80% of cases, with the underlying architecture involving chromosomal aberrations, single‐gene mutations, non‐coding variants, and oligogenic inheritance patterns.^5^ While individually uncommon, these conditions collectively affect millions of children worldwide.^6^ Traditional clinical decision‐support tools—which typically rely on static scoring systems or generalized population guidelines—are often ill‐equipped to capture such time‐varying complexity,^7^ underscoring the urgent need for more dynamic and personalized approaches.

### The AI paradigm shift

AI offers a new paradigm to address these challenges. Of particular relevance is machine learning (ML), wherein algorithms ‘learn’ to identify patterns directly from data rather than being explicitly programmed with deterministic rules. This approach is epitomized by deep learning (DL), which employs multi‐layered neural networks adept at modeling the non‐linear relationships concealed within vast, heterogeneous datasets—from medical imaging and physiological time‐series to genomic sequences. The ultimate objective is to leverage this capability to transition from broad, categorical diagnoses towards granular patient stratification, enabling precise, individualized care pathways and thereby realizing the ambition of precision medicine. In pediatrics, this capacity to model developmental trajectories and integrate sparse data from rare conditions holds particular promise for overcoming the limitations of adult‐derived models.

### Scope and literature search strategy

Translating this potential into pediatric practice requires a critical appraisal of the current evidence. A literature search was conducted in PubMed from January 2018 to December 2024, using search terms including combinations of (“artificial intelligence” OR “machine learning” OR “deep learning”) AND (“pediatric” OR “pediatric” OR “child*” OR “infant*” OR “neonate*”). The search was supplemented by a manual review of reference lists from key articles and recent high‐impact publications in the field. Foundational methodological works on explainable AI and causal inference^51^, ^55^ were also included as they informed the conceptual framework of this review. Given the narrative nature of this review, formal systematic screening procedures were not employed; rather, studies were selected to provide in‐depth coverage of three subspecialties: pediatric critical care, perinatal and neonatal medicine, and precision oncology.

**FIGURE 1:**
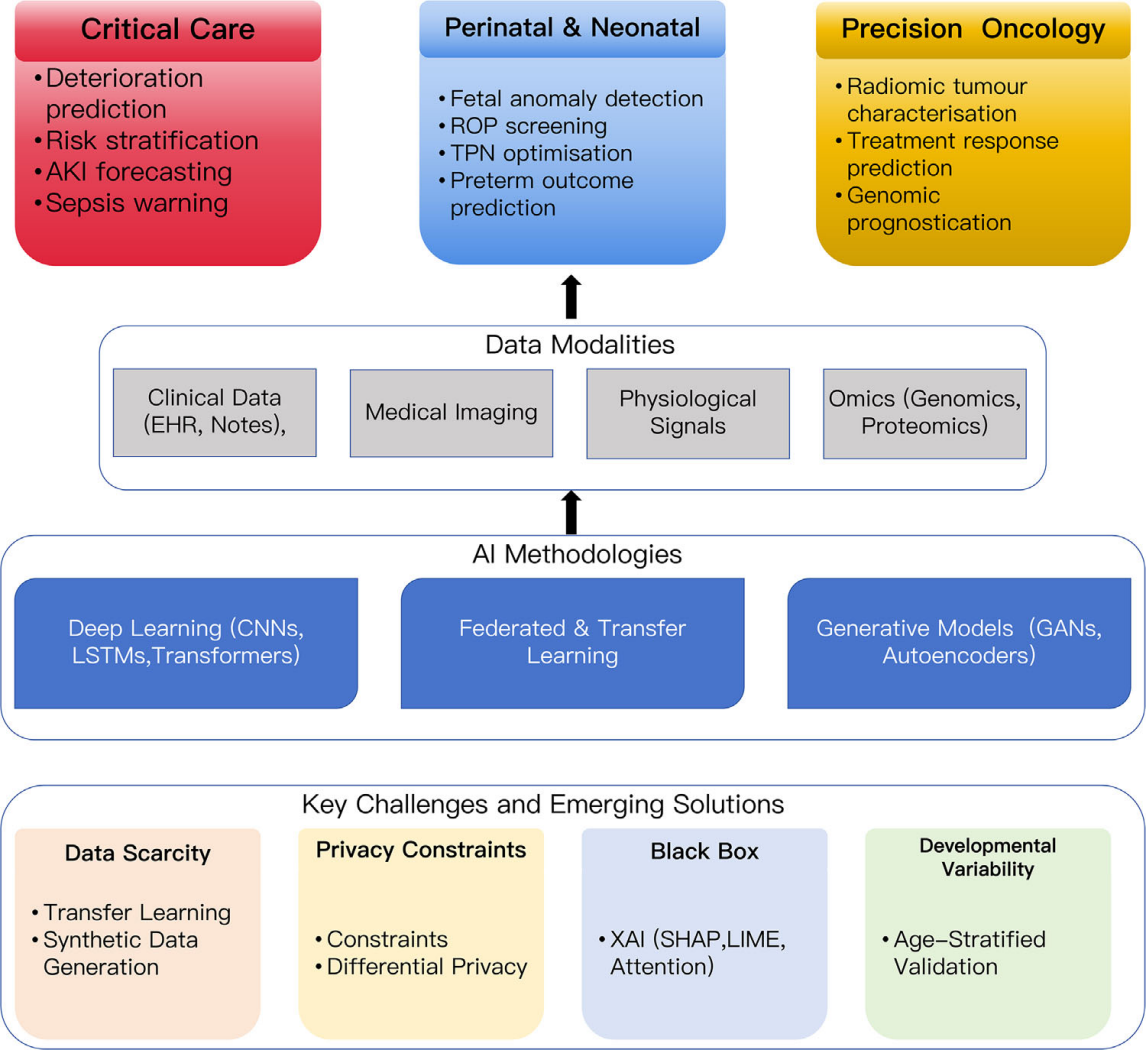
Translational framework for artificial intelligence applications in pediatric medicine. The framework illustrates three clinical domains with the most substantial evidence base and proximity to clinical translation (top panel), the heterogeneous data modalities and artificial intelligence (AI) methodologies employed across these domains (middle panel), key challenges impeding clinical translation alongside emerging solutions (challenge panel), and the pathway from algorithmic development to clinical implementation (bottom panel). AKI, acute kidney injury; CNNs, convolutional neural networks; EHR, electronic health records; GANs, generative adversarial networks; LIME, local interpretable model‐agnostic explanations; LSTMs, long short‐term memory networks; ROP, retinopathy of prematurity; SHAP, SHapley Additive exPlanations; TPN, total parenteral nutrition; XAI, explainable artificial intelligence.

While individual topics addressed herein have been reviewed elsewhere, existing reviews have typically focused on single subspecialties or isolated methodological challenges. This review aims to provide an integrated perspective, organized around a translational maturity framework. We selected critical care, perinatal medicine, and oncology as the three domains with the most substantial evidence base and proximity to clinical translation; other promising areas—including pediatric cardiology, neurology, and infectious diseases—warrant dedicated future reviews. The application of AI across these domains spans a spectrum of maturity: from pediatric intensive care unit (PICU) deterioration prediction, which has progressed to benchmarking against established clinical tools, to multiomics‐based prognostication and causal inference, which remain exploratory. A summary of the key literature is presented in Table S1. Figure 1 provides a schematic overview of the translational framework employed in this review. We begin by examining the current clinical applications across the three selected subspecialties, beginning with predictive analytics in critical care—the domain with the most mature evidence base—and then progressing to perinatal medicine and precision oncology.

## CLINICAL APPLICATIONS IN PEDIATRIC MEDICINE

Having established the rationale for focusing on critical care, perinatal medicine, and oncology, we examined the current state of AI applications within each domain. Table 1 provides an overview of the primary AI techniques, data modalities, key applications, and clinical readiness levels across these three subspecialties.

**TABLE 1:** Summary of artificial intelligence (AI) applications across pediatric subspecialties

Clinical domain	Primary AI techniques	Data types	Key applications	Validation status	Clinical readiness
Pediatric critical care (PICU)	LSTM networks; Transformer models; Autoencoders; Natural language processing	High‐frequency physiological time‐series; Electronic health records; Radiology reports; Infant movement videos	Dynamic mortality prediction; Real‐time deterioration forecasting; Acute kidney injury prediction (up to 48 h pre‐onset); Child abuse detection; Cerebral palsy prediction	Multi‐center external validation emphasized as the gold standard; the majority remain single‐center retrospective	Benchmarking against PRISM‐III/PIM3; prospective trials needed
Perinatal & neonatal medicine	Deep learning; Multiomics integration (genomics, proteomics, and epitranscriptomics)	Fetal ultrasonography; Retinal fundus images; Electronic health records; Total Parenteral Nutrition data	Fetal anatomical plane identification; Biometric measurement; Congenital anomaly screening; ROP severity grading; TPN optimization (TPN2.0)	TPN2.0: *R* = 0.94 correlation with expert decisions; most ML models show a high risk of bias, lack external validation	Early developmental stage; significant safety and ethical considerations
Pediatric oncology	Radiomics; Deep learning; Deep transfer learning; Generative AI	MRI; CT imaging; Genomic and epigenetic data; Single‐cell and bulk transcriptomics	Non‐invasive tumor characterization (digital biopsies); Tumor segmentation; Hepatic tumor differentiation; Treatment response prediction; Body composition analysis; Synthetic CT generation; Prognostic biomarker discovery	High accuracy in specific tasks (e.g., hepatic tumor differentiation); dependent on overcoming data challenges	Emerging; requires resolution of foundational data challenges

Abbreviations: AI, artificial intelligence; CT, computed tomography; LSTM, long short‐term memory; ML, machine learning; MRI, magnetic resonance imaging; PICU, pediatric intensive care unit; PIM3, Pediatric Index of Mortality 3; PRISM‐III, Pediatric Risk of Mortality III; R, correlation coefficient; ROP, retinopathy of prematurity; TPN, total parenteral nutrition.

### Predictive analytics in pediatric critical care

The PICU is a data‐intensive environment where the timeliness of intervention is paramount. AI, and DL in particular, offers the potential to shift risk stratification away from static, admission‐based assessments towards dynamic, real‐time forecasting of clinical deterioration.

#### The shift from static to dynamic prediction

Conventional PICU risk scores, such as the Pediatric Risk of Mortality III and the Pediatric Index of Mortality 3 (PIM3), are calculated from data acquired within the initial hours of admission. While PIM3 achieves respectable predictive performance (AUC of 0.88), these scores are constrained by fundamental limitations.^8^ First, they provide only a static snapshot, failing to account for the dynamic evolution of a patient's condition. Second, this design contributes to calibration drift; older scores such as PIM2 have been shown to overpredict mortality in contemporary cohorts.^8^ Most critically, these scores perform poorly when predicting outcomes beyond in‐hospital mortality, such as long‐term functional morbidity.^9^ This inadequacy presents a compelling opportunity for AI to provide continuous, multifaceted risk assessments that evolve with a patient's clinical trajectory.

Deep learning architectures designed for sequential data—including long short‐term memory (LSTM) networks and transformer models—are particularly suited to this task, enabling a shift from single‐point risk scores towards continuous, patient‐specific risk trajectories. Their technical foundations, validation requirements, and interpretability challenges are elaborated below.

#### Emerging clinical applications

Deep learning models have demonstrated impressive performance in pediatric diagnostics and prognostics, often surpassing traditional scores. AI frameworks analyzing millions of pediatric electronic health records have shown potential for automated, population‐level screening, although these findings await external validation.^10^ Natural language processing applied to radiology reports can detect subtle signs of childhood physical abuse with high accuracy.^11^ In prognostics, externally validated models can now predict cerebral palsy from videos of infant movements with clinically meaningful accuracy,^12^ and multi‐center validated models can forecast acute kidney injury up to 48 hours before clinical manifestation.^13^ These capabilities do not merely predict outcomes—they create critical opportunities for pre‐emptive intervention that could alter a patient's life trajectory.

Complementing physiological monitoring, AI‐assisted multiomics approaches have demonstrated broader utility in complex acute inflammatory conditions. Nygaard et al.^14^ identified a 4‐protein diagnostic signature for multisystem inflammatory syndrome in children using proteomic analysis, while revealing pathogenic mechanisms involving apolipoproteins, coagulation factors, and complement cascade dysregulation. This promising biomarker panel requires prospective validation before clinical implementation. Such applications illustrate the potential of AI‐driven biomarker discovery to extend beyond traditional diagnostic boundaries.

### AI in perinatal and neonatal medicine

The perinatal period presents a unique continuum of clinical challenges for which AI is providing powerful solutions. In prenatal care, DL models are being developed to automatically identify standard anatomical planes in fetal ultrasonography, perform biometric measurements, and screen for congenital anomalies—most notably cardiac defects—improving workflow efficiency and diagnostic consistency, although most models remain at the proof‐of‐concept stage with internal validation only. In the neonatal sphere, a prime example is retinopathy of prematurity (ROP) screening, where AI systems can automatically analyze retinal fundus images to quantify vascular changes and assign severity scores, improving diagnostic objectivity.^15^ This capability is particularly valuable for telemedicine platforms extending specialist expertise to underserved regions.

#### AI‐driven clinical decision support

Beyond diagnostics, AI is increasingly being applied to optimize clinical decision‐making in the neonatal intensive care unit (NICU). A notable externally validated clinical innovation is TPN2.0, an AI system trained on a decade of electronic health records on total parenteral nutrition (TPN) in the NICU. This model identified 15 standardized TPN formulations that could be allocated to neonates based on real‐time clinical data, achieving a strong correlation with expert decisions (*R* = 0.94) on external validation and demonstrating potential to enhance safety and reduce morbidities such as necrotizing enterocolitis.^16^

#### Challenges in neonatal AI

The application of AI within the NICU faces unique constraints. Neonates, particularly preterm infants, represent a highly vulnerable population where rapid physiological changes demand models capable of adapting to extreme dynamic shifts. These population‐specific hurdles, alongside broader issues of model validation and data ethics,^17^ are examined below.

### Advancing precision oncology in childhood cancers

AI is emerging as a critical enabling technology for precision oncology in childhood cancer, with applications spanning from non‐invasive diagnosis to tailored therapy design.^18^

#### Radiomics for non‐invasive characterization

Radiomics leverages AI to extract quantitative features from routine medical images, presenting a non‐invasive alternative to tissue biopsy. These ‘digital biopsies’ can predict tumor histology, grade, and underlying genomic mutations. For instance, a magnetic resonance imaging (MRI)‐based DL model demonstrated high accuracy in differentiating hepatic hemangioma from hepatoblastoma in infants in a single‐center retrospective study, although external validation is needed before clinical adoption.^19^ A prerequisite for robust radiomics is accurate tumor segmentation—a task for which DL has shown remarkable proficiency.^20^

#### Predicting treatment response and personalizing therapy

A central goal of precision oncology is tailoring therapy to individual profiles. Deep learning‐based analysis of body composition from routine computed tomography (CT) scans in pediatric lymphoma patients can identify individuals at high risk for treatment‐related complications, enabling proactive interventions; however, these findings derive from retrospective analyses and require prospective validation.^21^ Generative AI models are also being employed to synthesize CT images from MRI data, facilitating adaptive radiotherapy planning, although clinical implementation studies are ongoing.^22^

#### Genomic and epigenomic prognostication

Machine learning has proven adept at discerning prognostic patterns within high‐dimensional genomic and epigenetic data. By interrogating thousands of gene expression levels simultaneously, AI can uncover molecular signatures with superior predictive power compared to traditional markers. Advanced methodologies such as deep transfer learning can integrate single‐cell and bulk transcriptomic data to assign prognostic information to specific cellular subtypes, identifying the cell populations driving high‐risk phenotypes.^23^ These methodological advances are promising but remain largely exploratory, with clinical utility yet to be established through prospective studies. These AI‐derived biomarkers can inform refined risk stratification and guide personalization of treatment intensity.

In summary, the evidence base across pediatric oncology AI applications varies considerably. Federated learning approaches for brain tumor analysis have achieved multi‐center validation across 19 international sites, demonstrating the feasibility of privacy‐preserving collaboration. In contrast, most radiomic and genomic prognostication studies remain at the internal validation stage. Realizing the clinical potential of these applications requires addressing both the technical challenges of model development and validation and the foundational data challenges inherent to pediatric research (discussed in the following sections).

## TECHNICAL FOUNDATIONS AND METHODOLOGICAL CONSIDERATIONS

The clinical applications described in the previous section are underpinned by a set of common computational methodologies. This section examines the DL architectures that enable these advances, alongside the critical challenges of validation and interpretability that must be addressed for successful clinical translation.

### Deep learning architectures for clinical data

The interrogation of high‐frequency physiological data—characterized by high dimensionality and complex non‐linear interactions—has necessitated the adoption of sophisticated computational methods.^24^

Deep learning architectures designed for sequential data have proven particularly well‐suited to this challenge. LSTM networks evaluate each time point in the context of preceding data, capturing temporal dependencies within continuous physiological streams. This capability enables the prediction of not only global outcomes such as mortality but also the imminent onset of critical events, such as acute kidney injury, hours before clinical manifestation. A notable application is the use of routinely collected variables to construct dynamic models predicting adverse outcomes in preterm infants.^25^ Analogous approaches have been developed for biological sequence data, where pseudotime analysis methods construct trajectory models to capture dynamic cellular processes.^26^

However, LSTMs process sequences sequentially, which becomes computationally expensive for long time series—a limitation driving the development of attention‐based architectures.^27^, ^28^ Transformer models have consequently gained traction; their ‘self‐attention’ mechanisms identify long‐range dependencies without sequential processing constraints and have demonstrated superiority over LSTM baselines in clinical prediction tasks.^29^

Beyond sequential analysis, autoencoders are employed for unsupervised representation learning, reducing the dimensionality of complex multiomics data for integration into hybrid prognostic models.^30^ They also facilitate multi‐feature fusion, combining heterogeneous data sources—such as genomics, proteomics, and epitranscriptomic modifications—into unified representations for predicting adverse perinatal outcomes.^31^, ^32^, ^33^

### Validation and generalizability

Robust validation is the cornerstone of translating algorithmic performance into clinical trust. A crucial initial step is benchmarking against human experts to establish performance baselines and identify where AI might augment clinical judgment, particularly in tasks with high inter‐rater variability.^34^, ^35^

The most rigorous standard, however, is external validation: only by testing on independent, multi‐center datasets can confidence in a model's robustness be established.^12^, ^19^, ^35^, ^36^ This principle is equally critical in biomarker research, where expert consensus emphasizes that ‘large‐scale randomized controlled trials involving multiple centers are necessary to produce more reliable evidence‐based data.’^37^

Yet the current evidence base falls short of this standard. Furthermore, DL models are prone to overfitting on the relatively small datasets typical of pediatric research, and clinical prediction models often demonstrate illusory generalizability—performing well within development datasets but poorly out‐of‐sample.^38^, ^39^ A systematic review of ML models for predicting extubation failure in preterm neonates exemplifies this concern, finding that most studies were limited by a high risk of bias and lacked external validation.^40^

The preponderance of single‐center, retrospective studies carries additional risks: models trained on one demographic may systematically fail on underrepresented groups, thereby entrenching rather than alleviating healthcare disparities.^11^, ^22^, ^41^ This concern is amplified by findings that only 5% of large language model evaluations in healthcare used real patient data, with most relying on examination questions or clinician‐designed vignettes.^42^ The recent MedHELM (holistic evaluation of LLMs for medical tasks) framework, which evaluated nine frontier LLMs across 37 medical benchmarks, further demonstrated that models achieving near‐perfect scores on medical licensing examinations showed marked performance degradation in real‐world clinical tasks—particularly in quantitative domains such as medical calculations, billing code assignment, and structured query generation (scores of 0.53–0.63) compared to clinical note generation (0.74–0.85).^43^ This disparity between benchmark and real‐world performance underscores the inadequacy of examination‐based evaluation and highlights the need for task‐specific validation using authentic clinical data.

Future research must therefore pivot towards prospective, multi‐center trials that demonstrate not only algorithmic efficacy but also tangible improvements in patient‐centered outcomes.^36^, ^44^

### Model interpretability

A pervasive limitation across DL architectures is their ‘black box’ nature, wherein the reasoning underlying a prediction remains opaque.^45^, ^46^ This opacity poses a fundamental barrier to clinical adoption: clinicians are understandably reluctant to act upon recommendations they cannot interrogate or verify. The field of explainable AI has emerged in response to this challenge.^47^ This challenge extends beyond medicine; reviews of DL in biological taxonomy have similarly emphasized that lack of explainability undermines scientific trust.^47^

Techniques such as saliency mapping highlight which input features most strongly influence a model's output, providing post‐hoc rationales for individual predictions.^35^, ^48^ Attention weights in transformer architectures offer an intrinsic form of interpretability, revealing which time points or data elements the model prioritizes.

However, significant limitations persist. Post‐hoc explanations may not faithfully represent the model's true decision process, and the clinical utility of such explanations remains incompletely evaluated. Conventionally, the tension between model complexity and interpretability has been framed as a fundamental trade‐off: simpler, more interpretable models were assumed to sacrifice predictive performance, while high‐performing DL models resist straightforward explanation—a belief that has been deeply ingrained in the ML literature.^49^

A variety of explainable AI techniques have been developed to address interpretability, with the primary objective of fostering appropriate clinician confidence in algorithmic recommendations.^50^ Post‐hoc explanation methods attempt to explain predictions of complex ‘black box’ models after training.^45^ Prominent examples include SHAP (SHapley Additive exPlanations) values, which quantify the contribution of each input feature based on game‐theoretic principles;^51^ LIME (Local Interpretable Model‐agnostic Explanations), which generates locally faithful approximations using simpler surrogates;^52^ and saliency maps that highlight which image regions influenced classification decisions.^53^ Concept‐based explanations represent a more recent development, expressing model reasoning in terms of high‐level clinical concepts rather than raw features—potentially valuable in pediatrics where explaining predictions through recognizable clinical entities aligns more naturally with clinical reasoning.^54^

An alternative philosophy advocates for inherently interpretable models—algorithms transparent by design rather than requiring post‐hoc explanation, such as logistic regression, decision trees, and rule‐based systems.^55^ Importantly, this conventional trade‐off framing has been increasingly challenged. Rudin argues that for structured data with meaningful features, there is no evidence of a general accuracy‐interpretability trade‐off, and carefully designed interpretable models can achieve performance competitive with black‐box approaches in many clinical prediction tasks.^55^ Empirical evaluations have supported this view: recent benchmarking studies demonstrate that modern interpretable models, such as generalized additive models, can match or closely approach the predictive performance of complex black‐box models on tabular datasets typical of clinical applications.^55^, ^56^, ^57^ Thus, while the trade‐off may exist at the extremes, it is not an inherent property of the modeling problem—a nuance with important implications for pediatric AI, where clinical trust and accountability demand transparent decision‐making. For instance, in hepatocellular carcinoma prognostication, a transparent pathological scoring system demonstrated superior predictive performance compared to established systems in a nationwide multi‐center validation study.^58^

Addressing this challenge will require not only methodological advances but also empirical research into how clinicians interact with and respond to AI‐generated explanations—an essential step towards building the trust necessary for clinical integration.

## ADDRESSING FOUNDATIONAL DATA CHALLENGES

The development of robust AI models is contingent upon access to large, diverse datasets—a significant challenge in pediatric research, primarily due to the rarity of many childhood diseases.

### Overcoming data scarcity with transfer learning

The ‘data scarcity problem’ presents a formidable barrier to the application of AI in pediatrics,^11^, ^22^ further compounding the challenges of model validation and generalizability.

Transfer learning has emerged as a powerful strategy to address this limitation. This technique involves pre‐training a model on a large, often non‐pediatric dataset (e.g., adult data), allowing it to learn foundational features before being ‘fine‐tuned’ using smaller, task‐specific pediatric data. This approach is now widely adopted in pediatric medical imaging for tasks such as bone age assessment.^20^, ^59^ More sophisticated frameworks, such as Diagnostic Evidence GAuge of Single cells (DEGAS), utilize deep transfer learning to integrate single‐cell and patient‐level data, enabling knowledge transfer across different data modalities.^23^

### Advanced strategies: federated and few‐shot learning

Other advanced strategies address not only data scarcity but also patient privacy. Federated learning allows a shared model to be trained collaboratively across multiple institutions without centralizing sensitive patient data, offering a powerful solution to both data access and privacy challenges.^60^ Few‐shot learning directly targets extreme data rarity, developing models capable of generalizing from very small numbers of training examples—particularly relevant to the ‘long tail’ of rare pediatric diseases for which large datasets may never become available.^61^

### The role of generative AI and synthetic data

Generative AI, which encompasses models such as generative adversarial networks, offers another solution to data limitations. These models learn the underlying statistical distribution of a real dataset to generate novel synthetic data points that can augment small datasets, correct class imbalances, and enable wider data sharing without compromising patient privacy.

A compelling clinical application is the generation of synthetic CT images from MRI data for pediatric radiotherapy planning, which avoids exposing children to additional ionizing radiation while potentially creating shareable, fully anonymized datasets for research.^22^ However, ensuring the fidelity and diversity of synthetic data and robustly validating models trained upon it remains a significant technical hurdle.

## TOWARD ROBUST AND TRUSTWORTHY IMPLEMENTATION

The translation of AI from research into routine clinical practice requires surmounting significant scientific, ethical, and practical hurdles. The ultimate goal is to build trustworthy systems that can safely and equitably improve patient care.

Table 2 provides an overview of cross‐cutting methodological approaches and explainability techniques applicable across pediatric AI applications.

**TABLE 2:** Cross‐cutting methodological and explainable approaches in pediatric artificial intelligence (AI)

Approach category	Primary techniques	Data types	Key applications	Validation status	Clinical readiness
Cross‐cutting methodological approaches	Transfer learning; Federated learning; Few‐shot learning; Generative Adversarial Networks (GANs)	Adult‐to‐pediatric knowledge transfer; Multi‐institutional distributed data; Synthetic data	Bone age assessment; Privacy‐preserving collaborative training; Rare disease modeling; Synthetic CT for radiotherapy planning	Widely adopted in medical imaging; synthetic data fidelity validation ongoing	Increasingly implemented; privacy and data quality remain concerns
Explainable AI & causal inference	SHAP; LIME; Saliency maps; Concept‐based explanations; Inherently interpretable models; Causal ML	Model outputs; Feature attributions; High‐level clinical concepts	Model transparency; Fairness auditing; Therapeutic response estimation; Intervention effect analysis	Limited evaluation (only 1.2% of studies assessed calibration/uncertainty)	Critical for clinical trust; requires co‐development with clinicians

Abbreviations: AI, artificial intelligence; CT, computed tomography; GANs, generative adversarial networks; LIME, local interpretable model‐agnostic explanations; ML, machine learning; SHAP, SHapley Additive exPlanations.

### From prediction to causal inference

While explainable AI techniques can elucidate how predictions are made, true clinical utility requires a paradigm shift from correlation to causation. A major limitation of most current medical AI models is that they are purely predictive: they identify correlations within data but cannot infer causation. While such models can identify high‐risk patients, they cannot explain why—a limitation that fundamentally constrains their utility for guiding interventions. As Richens et al.^62^ demonstrate, the failure to distinguish genuine causal relationships from statistical associations may lead to clinically inappropriate or potentially harmful diagnostic conclusions.

Healthcare often requires information about cause–and–effect relations and counterfactual scenarios that purely predictive models cannot provide.^63^ Emerging causal ML approaches offer frameworks for estimating therapeutic responses, encompassing both beneficial effects and adverse reactions, which can inform pharmacological safety evaluations.^64^ Beyond prediction, causal ML provides tools for investigating how a system would respond to an intervention.^65^ There is a growing consensus to shift the focus of observational research in intensive care from prediction to causal inference.^66^

Recent advances in cancer biomarker discovery exemplify this paradigm: integration of methylome and transcriptome data enables identification of functionally relevant biomarkers whose mechanistic basis—such as promoter hypermethylation inversely correlating with tumor suppressor gene expression—provides biological plausibility beyond statistical association.^67^ In pediatrics, such approaches could enable models that not only predict which children are at high risk of deterioration but also estimate the likely benefit of specific interventions for individual patients.

For AI to translate into clinical value, model outputs must be integrated into workflows in a manner that is useful rather than burdensome. Optimal system design requires iterative co‐development with pediatric clinicians to ensure actionable, trustworthy information.^68^ The human factor remains critical, with clinician variability in trust and reliance shaping the ultimate utility of these tools.^50^ Furthermore, AI support depends on clinical context: a screening tool may require less detailed explanation than a system recommending specific therapeutic interventions.^69^

The path forward requires advancing both technical methods and empirical validation. AI has the potential to revolutionize neonatal intensive care by leveraging diverse data streams, with applications spanning imaging interpretation, prediction modeling, and real‐time monitoring.^70^, ^71^ Machine learning offers potential for more accurate risk stratification in NICU settings.^72^ However, prospective studies are needed to determine whether AI systems lead to better clinical adoption, fewer errors, and ultimately improved pediatric health outcomes.

### Digital twins: a paradigm for personalized and anticipatory care

An emerging paradigm is the ‘digital twin’—a dynamic, personalized computational model that mirrors an individual patient's physiological state in real time. Although digital twins for health remain in early stages, they hold great promise for revolutionizing healthcare systems.^73^ Digital twins are characterized by four critical features—explainability, intervenability, learnability, and diversability—aligning with the goals of P4 medicine (predictive, preventive, personalized, and participatory).^74^

In the neonatal context, a digital twin integrates continuous monitoring data (heart rate variability, oxygen saturation, and respiratory patterns) with patient‐specific parameters to create a virtual representation that evolves alongside its biological counterpart. This approach holds particular promise for the NICU, where rapid physiological changes demand adaptive, individualized models rather than population‐level predictions. Digital twins enable prospective simulation of interventions, allowing clinicians to explore ‘what if’ scenarios before implementation. Given that 40%–50% of medical errors are thought to be preventable,^75^ such simulation capabilities could prove valuable. Beyond immediate decision‐making, longitudinal digital twins could track developmental trajectories, providing early warning of deviations from expected milestones.

The state of the art in medical digital twin development is most advanced in oncology and cardiology.^76^ Nevertheless, implementation in neonatal care faces substantial hurdles: construction of accurate physiological models requires extensive domain expertise and validation; data integration from heterogeneous monitoring systems remains technically challenging; and computational demands of real‐time updating can be considerable. Ethical implications—concerning data ownership, consent, and potential algorithmic errors—also require careful consideration. A recent consensus statement noted that while digital twins were identified as a pivotal innovation for personalized healthcare, 87% of respondents opposed mandatory use, highlighting the need for patient‐centered implementation.^77^

### Developmental considerations unique to pediatric AI

Beyond the technical and ethical challenges common to all medical AI, pediatric applications face unique developmental considerations.^17^ Although data governance solutions—including federated learning and synthetic data generation,^60^, ^78^ can help address data scarcity, the unique developmental context of children—their continuous physiological change—means a single model is rarely appropriate for all ages. Unlike adult medicine, where physiological parameters remain relatively stable, pediatric patients undergo rapid and non‐linear changes in anatomy, physiology, and metabolism from birth through adolescence. Models must be developed and validated for specific age ranges, as exemplified by DL tools designed to track brain segmentation consistently across the lifespan.^79^

This developmental heterogeneity presents several challenges. First, training data must adequately represent each developmental stage; a model trained predominantly on school‐age children may perform poorly on neonates or adolescents. Second, normative reference ranges shift continuously—what constitutes a normal heart rate or respiratory pattern varies dramatically with age. Third, disease manifestations themselves are age‐dependent; sepsis presents differently in a premature infant compared to a teenager.

Furthermore, longitudinal prediction in pediatrics must account for expected developmental trajectories. A model predicting neurodevelopmental outcomes in preterm infants, for example, must distinguish pathological deviation from normal variation in developmental timing. This requires not merely cross‐sectional accuracy but the ability to model dynamic trajectories over months or years.

These developmental considerations highlight fundamental differences between pediatric and adult AI that extend beyond model recalibration. Adult AI benefits from larger training datasets and stable physiological baselines, whereas pediatric AI must contend with limited data, age‐dependent reference ranges, and continuously shifting normative values. The disease spectrum also diverges: conditions unique to children (e.g., ROP and congenital anomalies) and age‐dependent disease manifestations (e.g., neonatal versus adult sepsis) render adult‐trained models unreliable in pediatric settings. Furthermore, outcome metrics differ fundamentally—while adult AI often prioritizes short‐term mortality, pediatric applications must consider long‐term neurodevelopmental trajectories and quality‐adjusted life years across decades. These distinctions argue against uncritical adaptation of adult AI tools and mandate the development of pediatric‐native systems designed to accommodate the unique characteristics of children.

Addressing these challenges will require age‐stratified model development, pediatric‐specific training datasets, and validation protocols that explicitly assess performance across developmental stages.

### Navigating the regulatory and economic landscape

Clinical translation of AI is governed by regulatory frameworks for Software as a Medical Device (SaMD).^80^, ^81^ AI and ML algorithms present unique regulatory challenges as their performance can evolve post‐deployment through continuous learning. The U.S. Food and Drug Administration has developed a lifecycle‐based approach, including a ‘predetermined change control plan’ that allows manufacturers to prospectively define anticipated modifications via SaMD prespecifications and an algorithm change protocol, enabling agile improvements without full re‐review for every iteration.^80^

Significant challenges persist. Many AI algorithms function as ‘black boxes’, and real‐world performance can be inferior to initial evaluations.^80^ Many approved AI/ML devices have been validated using only retrospective studies,^81^ and systematic reviews consistently find poor external validity when models are applied to new populations.^40^

A recent systematic review of 519 studies evaluating large language models in healthcare revealed a profound disconnect between evaluation practices and the requirements for safe clinical deployment.^42^ While 95.4% of studies assessed accuracy, only 15.8% evaluated fairness, bias, or toxicity; only 4.6% addressed deployment considerations such as cost, latency, and infrastructure; only 1.2% evaluated calibration and uncertainty; and merely 5% utilized real patient care data rather than examination questions or synthetic vignettes.^42^ Such disconnects between validation and real‐world performance underscore the importance of Good Machine Learning Practice and robust post‐market monitoring.^80^

Demonstrating health economic value is equally critical for adoption.^82^, ^83^ AI tool value must be substantiated through rigorous cost‐effectiveness analyses. For example, an Australian analysis found that AI‐based screening for diabetic retinopathy would be highly cost‐saving by preventing blindness and saving hundreds of millions of dollars, despite increasing initial direct medical costs.^84^ However, a viable reimbursement pathway is essential. Models in the United States include per‐use payments via Current Procedural Terminology codes or technology add‐on payments from payers.^82^ Without a clear evidence and reimbursement strategy, securing institutional investment remains a formidable barrier.^82^, ^83^

### Ethical and governance considerations

Pediatric AI raises ethical challenges beyond those in adult populations. Consent frameworks must accommodate parental decision‐making alongside age‐appropriate child assent, with particular complexity in longitudinal applications where data collected in infancy informs care decisions years later. Data governance requires heightened protection given children's vulnerability, addressing prolonged retention periods, transition of data stewardship at adulthood, and re‐identification risks in rare diseases.

Algorithmic fairness demands explicit attention: underrepresentation of children—particularly from minority backgrounds or with rare conditions—in training datasets risks amplifying healthcare disparities. Accountability frameworks must clarify responsibilities among algorithm developers, institutions, and clinicians when AI‐assisted decisions contribute to adverse outcomes.

Policy priorities include developing pediatric‐specific regulatory guidance for AI medical devices, incentivizing representative pediatric dataset creation, and establishing standards for clinician training in AI‐assisted care. Addressing these challenges is essential for building the parental and societal trust upon which successful pediatric AI implementation depends.

## CONCLUSION AND FUTURE PERSPECTIVES

AI possesses transformative potential to reshape pediatric medicine. Across the three subspecialties examined in this review, AI models are demonstrating a capacity to analyze complex data with speed and accuracy that can augment clinical decision‐making. However, the strength of supporting evidence varies considerably. In some domains—such as PICU deterioration prediction and automated movement analysis for cerebral palsy detection—models have undergone rigorous external validation and approach clinical readiness. In others—including radiomic tumor characterization and multiomics‐based prognostication—the evidence remains largely exploratory, derived predominantly from single‐center retrospective analyses.

This review has highlighted progress across the field: from dynamic, real‐time prediction in critical care to enhanced prenatal diagnostics and the emergence of personalized therapeutic strategies in neonatology and oncology. Notably, applications with multi‐center external validation—such as federated learning for pediatric brain tumors and AI‐guided parenteral nutrition—offer the most immediate translational potential, while promising innovations in genomic prognostication and causal inference require further prospective validation before clinical adoption.

However, the journey from validated algorithm to trusted clinical tool is protracted and necessitates concerted, multidisciplinary effort.^85^ The future of pediatric AI will depend on several key priorities. First, establishing large‐scale, multi‐institutional collaborations is essential to curate the diverse datasets needed to train robust and equitable models; methodologies such as federated learning will be critical in this endeavor. Second, the research focus must evolve beyond pure prediction to encompass causal inference and explainability, thereby creating tools that clinicians can interrogate and trust to guide interventions. Third, all technological progress must be governed by robust ethical and regulatory frameworks designed specifically to safeguard the interests of children.^17^ Fourth, the development of standardized, pediatric‐specific evaluation frameworks is essential. Analogous to recent initiatives such as MedHELM for general medical AI,^43^ such frameworks must ensure that algorithms are rigorously assessed across the full spectrum of clinical tasks pertinent to child health—including age‐appropriate diagnostic reasoning, developmental milestone assessment, and weight‐based medication dosing—rather than relying solely on performance metrics derived from adult‐oriented benchmarks.

The ultimate goal is not to replace clinicians but to create intelligent systems that function as reliable, synergistic partners in care. As demonstrated in antimicrobial resistance research, AI methods show promise in guiding and accelerating the integration of scientific evidence, offering data‐driven support for translating research findings into actionable policies and clinical practice.^86^ To realize this vision, these systems must be more than merely accurate; they must be transparent, fair, and subject to continuous validation against real‐world outcomes. Critically, future research should prioritize prospective, multi‐center validation studies and implementation trials that assess real‐world clinical utility rather than retrospective model performance alone. By adhering to these principles, the medical community can responsibly harness the power of AI to improve the health and well‐being of every child.

## CONFLICT OF INTEREST

The authors declare no conflict of interest.

## Supporting information



Supporting Information
