# Supplementary material for: The transformative potential of artificial intelligence in pediatric medicine: Current applications, methodological challenges, and future directions
Source: Pediatr Investig. 2026 May 1:10.1002/ped4.70061. Online ahead of print. doi: 10.1002/ped4.70061 (PMC13399193; doi:10.1002/ped4.70061)
Supplement: Supplementary file 1 — Supporting Information [file PED4-9999-0-s001.pdf]

Supplementary Material for

The Transformative potential of artificial intelligence in pediatric medicine: Current applications, methodological challenges, and future directions

Ruisong Wang, Xiaoman Ding, Wanyue Zhang, Tieliu Shi

Table S1 Summary of cited literature on AI in pediatric medicine

Area of pediatrics	First author & year	Technique	Primary application	Validation status	Key contribution to this review
Pediatric nephrology	Dong et al. (2021)	Machine Learning (Ensemble)	Prognosis	External validation (multi-center)	Multi-center model predicting acute kidney injury up to 48 hours before onset.
Pediatric critical care medicine	Liang et al. (2019)	Deep Learning	Triage/Diagnosis	Internal validation (single-center)	Broad diagnostic triage tool for common pediatric conditions using a very large EHR dataset.
Neonatology	Feng et al. (2021)	Deep Learning (LSTM)	Prognosis	Internal validation (single-center)	Predicts mortality risk in preterm infants using time-series vital sign data.
Neonatology	Groos et al. (2022)	Deep Learning (CNN)	Diagnosis	External validation	Predicts cerebral palsy from videos of infant movements with high accuracy on external validation.
Neonatology	Phongpreecha et al. (2025)	Deep Learning (Transformer)	Therapeutic Guidance	External validation	AI-guided system (TPN2.0) to create and assign personalized parenteral nutrition formulas; validated externally with high correlation to expert decisions (R=0.94).
Pediatric immunology	Nygaard et al. (2024)	AI-assisted Proteomics	Diagnosis	Internal validation	Identifies a 4-protein diagnostic signature for Multisystem Inflammatory Syndrome in Children (MIS-C).
Neonatology/ ophthalmology	Sabri et al. (2022)	Deep Learning	Diagnosis	Narrative review	AI-based screening for retinopathy of prematurity (ROP) via automated retinal fundus image analysis

Neonatology	Hoff Calegari et al. (2025)	Machine Learning (Systematic Review)	Prognosis	Systematic review	Systematic review of ML models predicting extubation failure in preterm neonates
Neonatology	Keles & Bagci (2023)	AI/Deep Learning	Overview/Monitoring	Systematic review	Human-in-the-loop AI systems for NICU monitoring and risk classification
Neonatology	Sullivan et al. (2024)	AI/ Machine Learning	Overview	Conceptual framework	Framework for transforming neonatal care with AI, addressing challenges and opportunities
Neonatology	Sullivan et al. (2025)	Machine Learning	Prognosis	Su'mInternal validation (comparative)	Team-based competition comparing ML models for NICU mortality prediction using clinical variables and vital signs; logistic regression (AUC 0.818) outperformed complex models, emphasizing interpretability over complexity.
Pediatric Oncology	Florkow et al. (2020)	Generative AI (CNN)	Therapeutic Guidance	Internal validation (single-center)	Generates synthetic CT scans from MRI for adaptive radiotherapy planning in pediatric patients.
Pediatric Oncology	Tram et al. (2023)	Deep Learning	Prognosis	Internal validation (single-center)	Automated CT analysis of body composition to predict late treatment effects in lymphoma.
Pediatric Oncology	Yang, Zhou & Li (2023)	Deep Learning (CNN)	Diagnosis	External validation (multi-center)	MRI-based model to differentiate between two common pediatric liver tumors (HH and HBL).

Pediatric Neuro-oncology	Lee et al. (2024)	Federated Learning (3D-UNet)	Joint tumor classification and segmentation	External validation (multi-center federated)	Demonstrates privacy-preserving multi-institutional collaboration across 19 international sites; FL achieves comparable performance to centralised training (<1.5% decrease in classification, 3% in segmentation) while addressing data scarcity in pediatric neuro-oncology
Radiology	Beheshtian et al. (2023)	Deep Learning	Diagnosis/Ethics	Bias analysis study	Demonstrates significant algorithmic bias (sex, age, maturity) in a pediatric bone age model.
Radiology	Choi et al. (2022)	Deep Learning (CNN)	Diagnosis	External validation (multi-center)	AI model for detecting pediatric skull fractures that improves clinician performance.
Radiology	E et al. (2019)	Deep Learning	Image Segmentation	Internal validation (single-center)	Accurate segmentation of lungs on pediatric chest X-rays to improve pneumonia classification.
Pediatric Radiology	Mayourian et al. (2024)	Deep Learning	Diagnosis	External validation (multi-center)	AI-ECG algorithm that outperforms a pediatric cardiologist in detecting left ventricular hypertrophy.
Neuroimaging	Simarro et al. (2024)	Deep Learning (3D U-Net)	Image Segmentation	External validation (multi-center)	A single model for robust brain segmentation across both pediatric and adult populations.
Radiology	Summers (2018)	Deep Learning (Commentary)	Diagnosis	Commentary	Discusses a mature application of DL (bone age) and introduces saliency maps for interpretability.

Radiology	Suh et al. (2023)	Deep Transfer Learning	Diagnosis	External validation (single-center)	Transfer learning approach for pediatric bone age assessment
Methodology	Johnson et al. (2022)	Deep Transfer Learning	Data Integration	Methodological framework	Details the DEGAS framework for integrating single-cell and bulk transcriptomic data.
General Pediatrics	Malhotra et al. (2023)	AI/ML (Editorial)	Overview	Editorial	Provides a high-level perspective on the emerging role and future challenges of AI in pediatrics.
Methodology	Poirion et al. (2021)	Deep Learning (Autoencoder)	Data Integration	Methodological framework	Describes a deep learning method for integrating clinical and multiomics data for prognostication.
Emergency Medicine	Shahi et al. (2021)	Deep Learning & NLP	Diagnosis	Internal validation (single-center)	Model combining clinical data and NLP of radiology reports to detect child physical abuse.
Neonatology/ ethics	Boch et al. (2022)	N/A (Ethical Framework)	Ethics	Ethical framework	Ethical considerations for AI application in vulnerable neonatal populations

AI, artificial intelligence; CNN, convolutional neural network; CT, computed tomography; DEGAS, diagnostic evidence gauge of single cells; DL, deep learning; ECG, electrocardiogram; EEG, electroencephalogram; HER, electronic health record; FAST, focused assessment with sonography for trauma; HBL, hepatoblastoma; HH, hepatic hemangioma; LSTM, long short-term memory; MIS-C, multisystem inflammatory syndrome in children; ML, machine learning; MRI, magnetic resonance imaging; NLP, natural language processing; TPN, total parenteral nutrition; VNN, variational neural network.

Note: This table summarises primary research applying AI/ML methods to pediatric populations. Foundational methodological works on causal inference and explainable AI (e.g., Rudin, 2019; Lundberg & Lee, 2017) are cited in the main text but not included here as they are not pediatric-specific.
